# Limited utility of imaging-based (N) markers in predicting cerebral spinal fluid Alzheimer’s disease biomarker positivity in lecanemab-eligible mild cognitive impairment

**DOI:** 10.3389/fneur.2025.1735548

**Published:** 2025-12-04

**Authors:** Takeshi Kuroda, Yukiko Mori, Daiki Shoji, Satomi Kubota, Moeko Noguchi-Shinohara, Kenjiro Ono, Hidetomo Murakami

**Affiliations:** 1 Department of Neurology, Showa Medical University School of Medicine, Tokyo, Japan; 2 Department of Neurology, Showa Medical University Fujigaoka Hospital, Kanagawa, Japan; 3 Department of Neurology, Kanazawa University Graduate School of Medical Sciences, Kanazawa, Japan

**Keywords:** Alzheimer’s disease, mild cognitive impairment, anti-amyloid β therapy, lecanemab, imaging-based (N) markers

## Abstract

**Background:**

Early detection of mild cognitive impairment (MCI) due to Alzheimer’s disease (AD) is essential for the timely initiation of anti-amyloid β (Aβ) therapy. In clinical practice, cerebrospinal fluid (CSF) testing and amyloid positron emission tomography (PET) are not feasible for all patients; therefore, initial screening sometimes relies on imaging-based neurodegeneration (N) markers within the amyloid-tau-neurodegeneration AT(N) framework. However, the diagnostic accuracy of imaging-based (N) markers remains uncertain in real-world MCI cohorts being evaluated for anti-Aβ antibody therapy. We aimed to assess the utility of imaging-based (N) markers in predicting AD pathology in patients with MCI who may be eligible for lecanemab.

**Methods:**

Thirty-six patients with MCI who were potentially eligible for lecanemab underwent CSF biomarker testing and were subsequently classified as MCI unlikely due to AD (MCI non-AD; *n* = 14) or MCI due to AD (MCI-AD; *n* = 22). Demographics, general risk factors, neuropsychological test scores, and imaging-based (N) markers—medial temporal atrophy (MTA) on magnetic resonance imaging (MRI) and regional cerebral blood flow (CBF) reductions on single-photon emission computed tomography (SPECT)—were compared between groups.

**Results:**

MCI-AD was diagnosed in 22 patients (61%). Demographics, neuropsychological test scores, and most general risk factors did not differ between groups, except for diabetes, which was more frequent in the MCI non-AD group. Overall, 34 of 36 patients (94%) were classified as (N) + based on MRI or SPECT (91% MCI-AD; 100% MCI non-AD). There were no significant differences in mean MTA scores or the degree of CBF reduction between groups. In contrast, the proportion of MRI (N) + patients was significantly higher in the MCI non-AD group than in the MCI-AD group, and two patients with MCI-AD were (N) − on both MRI and SPECT.

**Conclusion:**

Relying on imaging-based (N) markers to select MCI patients who may be eligible for lecanemab prior to CSF biomarker testing may lead to inefficient diagnostic pathways and may fail to identify patients who could benefit from anti-Aβ therapy.

## 1 Introduction

The global prevalence of dementia is expected to increase from 57 million in 2019 to 152 million by 2050, representing a 166% increase (1). Alzheimer’s disease (AD), the most common cause of dementia, is a progressive neurodegenerative disorder. Once patients reach the dementia stage, they typically require long-term care and have a shortened life expectancy. A therapy targeting the amyloid-β (Aβ) protein, a key pathogenic factor in AD, was recently developed to slow disease progression (2). Lecanemab reduces the rate of clinical decline, potentially lowering long-term care costs and prolonging functional independence, whereas non-pharmacological interventions provide only modest symptomatic benefits and do not modify the underlying pathology (3). Early initiation of lecanemab delays cognitive decline, emphasizing the importance of accurately identifying patients with mild cognitive impairment (MCI) who exhibit AD pathology (2, 4).

Lecanemab has been available in Japan as an insurance-covered drug for MCI due to AD and mild AD dementia since December 20, 2023. Cerebrospinal fluid (CSF)-Aβ measurements and amyloid positron emission tomography (PET) are covered by insurance to confirm amyloid pathology in patients with MCI and mild dementia who may be eligible for anti-Aβ antibody therapy, based on optimal-use guidelines established by the Ministry of Health, Labor and Welfare in Japan.

Before the introduction of AD biomarkers into routine clinical practice, AD was diagnosed in Japan based on clinical symptoms, neuropsychological tests, magnetic resonance imaging (MRI), and cerebral perfusion single-photon emission computed tomography (SPECT). The amyloid-tau-neurodegeneration AT(N) classification system proposed by the National Institute on Aging–Alzheimer’s Association (NIA–AA) research framework defines AD in terms of its underlying pathologic processes (5). AD biomarkers are grouped into three categories: Aβ deposition (A), pathologic tau (T), and neurodegeneration or neuronal injury (N) (5, 6). While A and T biomarkers reflect AD-specific pathology, N biomarkers—such as elevated CSF total tau (7), hypometabolism on ^18^F-fluorodeoxyglucose (FDG)-PET (8), and atrophy on structural MRI (9–11)—indicate neurodegeneration or neuronal injury from various etiologies and are therefore not specific to AD, representing a recognized limitation of the N category. FDG-PET is not reimbursed under the Japanese national health insurance system for dementia care; thus, cerebral perfusion SPECT is routinely used in clinical practice. FDG-PET measures regional glucose metabolism, whereas SPECT assesses regional cerebral blood flow (CBF). Regional hypoperfusion in the temporal and parietal lobes, posterior cingulate gyrus, and precuneus may serve as surrogate (N) markers (12). A systematic review of imaging studies in AD dementia reported that FDG-PET has a sensitivity of 89% and specificity of 74%, whereas SPECT has a sensitivity of 64% and specificity of 83% for distinguishing pathologically confirmed AD from non-AD dementias (9).

CSF testing is invasive and PET is costly; therefore, these examinations are not feasible for all patients with MCI and may necessitate initial screening using conventional imaging biomarkers. However, the diagnostic accuracy of imaging-based (N) biomarkers is limited, and there is little evidence regarding how well these markers perform specifically in real-world MCI cohorts being evaluated for anti-Aβ antibody therapy such as lecanemab. Reliance on imaging-based screening may lead to inefficient diagnostic pathways and may result in failure to identify MCI patients who may be eligible for anti-Aβ therapy. Therefore, the present study aimed to evaluate the utility and potential risks of using conventional imaging-based (N) markers to select patients with MCI for further biomarker testing. Our primary objective was to determine whether imaging-based (N) markers can accurately identify AD pathology in MCI patients who may be eligible for lecanemab; we hypothesized that their predictive value would be limited.

## 2 Materials and methods

### 2.1 Study participants

This single-center retrospective observational study consecutively enrolled patients who visited the Memory Clinic of the Department of Neurology at Showa Medical University School of Medicine in Tokyo, Japan, between January 1 and December 31, 2024. We first excluded patients who were unable to complete the full baseline assessment, including brain MRI, SPECT, Clinical Dementia Rating (CDR), Mini-Mental State Examination (MMSE), Montreal Cognitive Assessment (MoCA), Frontal Assessment Battery (FAB), and blood tests. The same psychologist administered the CDR and neuropsychological tests, and detailed clinical information was obtained from caregivers.

**Figure 1:**
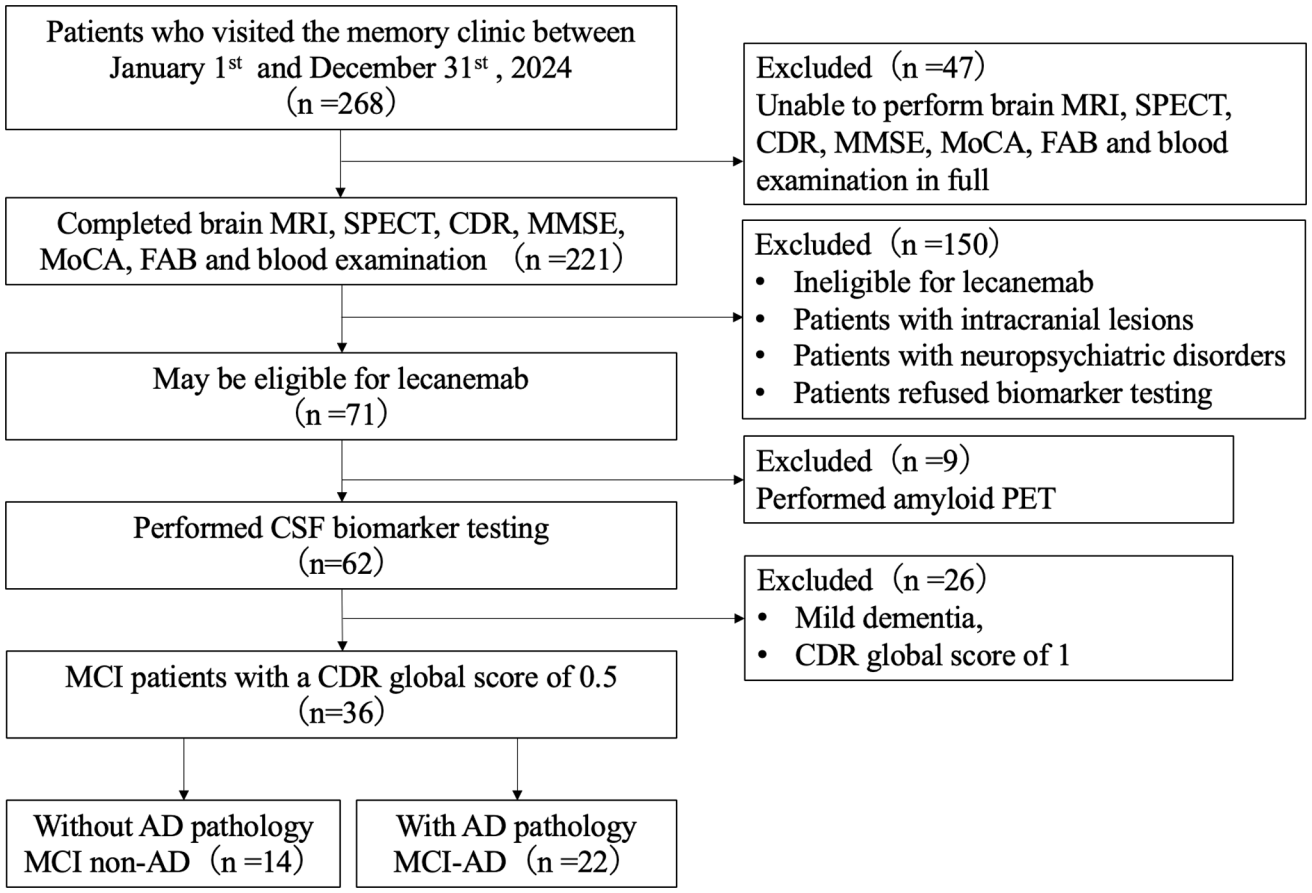
Patient recruitment flowchart. Patients with a CDR global score of 0.5 or 1, an MMSE score of ≥22, and no contraindications such as more than five cerebral microbleeds, a history of cerebral hemorrhage >1 cm, or cortical superficial siderosis were considered potentially eligible for lecanemab and underwent CSF biomarker testing or amyloid PET. MRI, magnetic resonance imaging; SPECT, single-photon emission computed tomography; CDR, Clinical Dementia Rating; MMSE, Mini-Mental State Examination; MoCA, Montreal Cognitive Assessment; FAB, Frontal Assessment Battery; CSF, cerebrospinal fluid; PET, positron emission tomography; MCI, mild cognitive impairment; AD, Alzheimer’s disease.

Patients with intracranial lesions such as vascular disorders or brain tumors, a history of head trauma, or neuropsychiatric disorders, as well as those who refused biomarker testing, were excluded. Among patients who completed all of these examinations, CSF or amyloid PET biomarker testing was performed as an insurance-covered examination in individuals who were potentially eligible for lecanemab; that is, patients with a CDR global score of 0.5 or 1, an MMSE score of ≥22, and no contraindications such as five or more cerebral microbleeds, a history of cerebral hemorrhage >1 cm, or cortical superficial siderosis (2). Of the patients who underwent CSF biomarker testing, those who met the clinical diagnostic criteria for MCI (13) and had a CDR global score of 0.5 (14) were ultimately included in this study (Figure 1).

Following CSF biomarker testing, patients with amyloid pathology were classified as “MCI due to AD—intermediate or high likelihood” (MCI-AD), and those without amyloid pathology were classified as “MCI unlikely due to AD” (MCI non-AD), according to established diagnostic criteria (15). We further classified MCI as amnestic (a-MCI) or non-amnestic (na-MCI) based on the MoCA results and compared the distributions of these subtypes between the MCI non-AD and MCI-AD groups (13). Patients who scored ≤2 in the delayed recall section of the MoCA were categorized as amnestic, whereas those who scored ≥3 were categorized as non-amnestic. Furthermore, a-MCI was classified as single-domain if only memory was impaired, or as multidomain if other cognitive domains—such as visuospatial abilities, executive function, language, or attention—were also affected. Similarly, na-MCI was categorized as single-domain or multidomain (13).

The Ethics Committee of Showa Medical University School of Medicine approved the study, which was conducted in accordance with the principles of the Declaration of Helsinki. All participants provided written informed consent. No formal sample size calculation was performed, as this study used a consecutive sampling design that included all eligible patients who underwent CSF biomarker testing during the study period. As an observational study without any intervention, randomization procedures were not applied. With the exception of the blinded visual assessment of medial temporal lobe atrophy on MRI, neither participants nor clinical evaluators were blinded, as no interventional allocation was involved.

### 2.2 MRI

Structural MRI was conducted using a 1.5 T MR scanner (Magnetom Essenza; Siemens, Munich, Germany). The protocol included fluid-attenuated inversion recovery, as well as diffusion-weighted, T2-weighted, susceptibility-weighted, and high-resolution three-dimensional (3D) T1-weighted (144 sagittal slices; 1.0 × 1.0 × 1.25 mm^3^) imaging. Diffusion-weighted and T2-weighted imaging, as well as fluid-attenuated inversion recovery, were used to identify abnormal cortical signals (such as encephalitis and Creutzfeldt–Jakob disease) and intracranial lesions such as cerebral infarction, hemorrhage, encephalitis, and brain tumors. Susceptibility-weighted imaging was applied to detect cerebral microbleeding and cortical superficial siderosis. The visual assessment of medial temporal lobe atrophy (MTA) was performed on coronal T1-weighted images by averaging scores for the left and right sides (range, 0–4), with high scores indicating great atrophy. Evaluation was conducted by two experienced neurologists under blinded conditions (16, 17). We confirmed MTA occurrence when the average MTA score met or exceeded the age-specific threshold based on recently proposed cutoffs for abnormal scores: ≥1 for individuals aged <65 years, ≥1.5 for those aged 65–74 years, and ≥2 for people aged ≥75 years (18). Furthermore, 3D T1-weighted images were analyzed using a voxel-based specific regional analysis system for Alzheimer’s disease (VSRAD Advance 2; Eisai, Japan) for the volumetric evaluation of MTA (19). The VSRAD software is used in the clinical diagnosis of AD in Japan. It enables the quantitative assessment of the extent of brain atrophy (percentage of volume reduction in gray and white matter) compared with that in an MRI database of 80 age-matched healthy controls, using voxel-based morphometry. The Z-score [(normal control average of voxel-level - patient’s voxel-level) / (normal control standard deviation)] is calculated in each voxel, and the areas with a Z-score ≥ 2 are considered atrophied. The extent of atrophy was expressed as percent volume loss. Using this software, we automatically calculated the percentage of medial temporal lobe (MTL) gray matter atrophy (%MTL-GM) and the extent to which MTL atrophy was selective for the whole brain (%MTL ratio) (20). High %MTL-GM and %MTL ratios indicated severe MTL atrophy.

### 2.3 SPECT and 3D stereotactic surface projection analysis

Cerebral perfusion SPECT data were obtained using three head gamma cameras (GCA-9300R; Canon Medical Systems, Tochigi, Japan) equipped with an ultra-high-resolution fan-beam collimator. SPECT images were acquired 15 min after injecting a 222 MBq dose of 123I-IMP (Nihon Medi-Physics Co. Ltd., Tokyo, Japan). The projection data were obtained for 20 min. The imaging parameters were as follows: matrix size = 128 × 128; pixel size = 2.9 mm; slice thickness = 2.9 mm; energy window = 159 keV ± 20%. Data were reconstructed using the filtered back-projection method with a Butterworth filter (0.1 cycle/pixel). The SPECT images were analyzed using 3D stereotactic surface projection (3D-SSP) to generate Z-score maps in the iSSP software included in “medi+FALCON version 1.2” (Nihon Medi-Physics Co., Ltd., Tokyo, Japan). The Z-score was used to detect the areas deviating from the database created from the data of multiple healthy individuals after anatomical standardization. To evaluate the CBF in the region of interest (ROI), this software utilizes Z-summation, an index based on the Z-score summation analysis method normalized by the ROI size (number of pixels). The parietal lobes (superior and inferior parietal lobules, angular gyrus, and supramarginal gyrus), posterior cingulate gyrus, and precuneus were set up as ROIs for AD, and the degree of deviation from the normal database was automatically evaluated via the 3D-SSP software using standard deviation (SD). Details of the 3D-SSP have been previously reported (21, 22). A large SD value indicates a great degree of CBF reduction in the ROI and is considered abnormal if it exceeds 1.64 SD. Of the six ROIs (left and right parietal lobes, posterior cingulate gyrus, and precuneus), we evaluated the number of regions regarded as abnormal.

### 2.4 General risk factors

Blood samples were assessed for lipid (low- and high-density lipoprotein cholesterol and triglycerides) and glucose (glycated hemoglobin) metabolisms. The presence of hypertension, hyperlipidemia, diabetes, atrial fibrillation or cardiac failure, current smoking status, and alcohol consumption were determined based on blood examination results, self-reported medical history, and medication use data.

### 2.5 CSF biomarker testing

The CSF was obtained by puncturing the lumbar region between the L3/L4, L4/L5, and L5/S1 intervertebral spaces with a 25-gage needle and syringe. Subsequently, the CSF was collected in polypropylene tubes (23). Aβ 1–42, Aβ 1–40, the Aβ 1-42/Aβ 1–40 ratio, and tau phosphorylated at threonine 181 (p-tau) were measured using the Lumipulse® system. The Lumipulse® system, manufactured by Fujirebio, is an automated chemiluminescent enzyme immunoassay platform designed for highly sensitive and specific measurement of biomarkers, including Aβ and p-tau, in the CSF (24). Details of the measurement method have been previously provided (25). The presence of AD pathology was assessed using the Aβ1-42/Aβ1-40 ratio and p-tau to determine the eligibility of lecanemab administration.

### 2.6 AT(N) classification

AD biomarkers were grouped into those of Aβ deposition (A), pathological tau (T), and neurodegeneration (N) (5, 6). We used the CSF Aβ1-42/Aβ1-40 ratio to determine whether patients were A– or A+; patients were defined as A + when the Aβ1-42/Aβ1-40 ratio was <0.067. CSF p-tau concentrations were used to define tau pathology, which was defined as T + when p-tau levels exceeded 59.0 pg/mL (24, 26). We defined (N) + as MTA on structural MRI, CBF reduction on cerebral perfusion SPECT, or both. We assessed the average scores of the right and left visual MTA using structural MRI. SPECT findings were defined as (N) + if at least one of the six ROIs (left and right parietal lobes, posterior cingulate gyrus, and precuneus) showed a reduction in CBF beyond the normal range (1.64 SD). Patients with MCI exhibiting an A– profile were considered unlikely to have AD-related pathology (MCI non-AD), whereas those with an A + profile were regarded as MCI due to AD (MCI-AD) (15).

### 2.7 Statistical analysis

We first inspected the distribution of continuous variables to confirm that the assumptions for parametric testing were reasonably met. Group comparisons between the MCI-AD and MCI non-AD groups were then performed for demographic variables, neuropsychological scores, MRI-based measures, SPECT indices, and CSF biomarkers. Unpaired t-tests were used to analyze differences in mean age, years of education, neuropsychological test scores, CDR scores, and MRI, SPECT, and CSF biomarker results between groups. Chi-square tests were conducted to examine sex distribution and the prevalence of general dementia risk factors in the MCI-AD and MCI non-AD groups.

All tests were two-tailed and performed using SPSS version 29.0.1.0 (IBM Corp., Armonk, NY, USA). Statistical significance was defined as *p* < 0.05. Results are presented as means and SDs. Given the limited sample size, multivariable modeling was not performed.

## 3 Results

### 3.1 Demographic and clinical characteristics

Thirty-six patients (mean age: 80.3 ± 3.7 years; 24 women) who met the clinical diagnostic criteria for MCI and had a CDR global score of 0.5 underwent CSF biomarker testing. Of these, 14 patients (38.9%) had no AD pathology, defined by a normal CSF Aβ1-42/Aβ1-40 ratio, and were classified as MCI non-AD (mean age: 80.1 ± 4.0 years; 7 women). The remaining 22 patients (61.1%) had AD pathology, indicated by a decreased CSF Aβ1-42/Aβ1-40 ratio, and were categorized as MCI-AD (mean age: 80.4 ± 3.6 years; 17 women).

The MCI-AD group had significantly lower CSF Aβ1-42 concentrations (674.1 ± 176.9 vs. 1,256.6 ± 385.2 pg./mL, *p* < 0.0001) and Aβ1-42/Aβ1-40 ratios (0.048 ± 0.010 vs. 0.097 ± 0.017, *p* < 0.0001), as well as significantly higher CSF p-tau concentrations (82.0 ± 32.6 vs. 41.5 ± 11.5 pg./mL, *p* < 0.0001), than the MCI non-AD group. No significant difference was observed in CSF Aβ1-40 levels between the groups (14,285.1 ± 3,529.3 vs. 13,026.9 ± 3,571.7 pg/mL, *p* = 0.32).

All patients had a CDR global score of 0.5, and there was no significant difference in the CDR sum of boxes (*p* = 0.23). No significant differences were observed between the groups in mean age (*p* = 0.84), percentage of women (*p* = 0.42), years of education (*p* = 0.73), percentage of smokers (*p* = 0.13), alcohol consumption (*p* = 0.42), hypertension (*p* = 0.79), dyslipidemia (*p* = 0.70), or atrial fibrillation/cardiac failure (*p* = 0.74). In contrast, the proportion of patients with diabetes was significantly higher in the MCI non-AD group (*p* = 0.049) (Table 1).

**Table 1:** Comparison between mild cognitive impairment unlikely due to Alzheimer’s disease and mild cognitive impairment due to Alzheimer’s disease.

Group (*n*)	MCI non-AD (14)	MCI-AD (22)	*p*-value
Age	80.1 ± 4.0	80.4 ± 3.6	0.84
Female (%)	7 (50)	17 (77.3)	0.42
Education year	14.1 ± 2.1	13.8 ± 2.1	0.73
CDR global score	0.5	0.5	1.00
CDR sum of the boxes	2.0 ± 1.2	2.5 ± 0.9	0.23
CSF biomarkers			
Aβ42 (pg/ml)	1256.6 ± 385.2	674.1 ± 176.9	< 0.0001*
Aβ40 (pg/ml)	13026.9 ± 3571.7	14285.1 ± 3529.3	0.32
Aβ42/40	0.097 ± 0.017	0.048 ± 0.010	< 0.0001*
p-tau (pg/ml)	41.5 ± 11.5	82.0 ± 32.6	< 0.0001*
General risk factors			
Smoking (%)	4 (28.6)	2 (9.1)	0.13
Alcohol (%)	7 (50)	8 (36.4)	0.42
Hypertension (%)	7 (50)	12 (54.5)	0.79
Dyslipidemia (%)	6 (42.9)	8 (36.4)	0.70
Diabetes (%)	5 (35.7)	2 (9.1)	0.049*
Cardiac disease (%)	1 (7.1)	1 (4.5)	0.74

MCI, mild cognitive impairment; AD, Alzheimer’s disease; CDR, clinical dementia rating; CSF, cerebral spinal fluid; Aβ, amyloid β; p-tau, tau phosphorylated at threonine 181. *indicates statistical significance at *p* < 0.05.

### 3.2 Neuropsychological test results

Total MMSE, MoCA, and FAB scores did not significantly differ between the groups (*p* = 0.32, 0.86, and 0.76, respectively). All patients were classified as having a-MCI and showed memory impairment, with scores <2 in the delayed recall section of the MoCA. One patient in the MCI-AD group was classified as a-MCI single-domain, whereas all other patients were classified as a-MCI multidomain (Table 2).

**Table 2:** Comparison of the proportion of amnestic and non-amnestic mild cognitive impairment and neuropsychological test results.

Group (*n*)	MCI non-AD (14)	MCI-AD (22)	*p*-value
na-MCI (%)	0 (0)	0 (0)	1.00
a-MCI (%)	14 (100)	22 (100)	1.00
Single domain (%)	0 (0)	1 (4.5)	0.42
Multidomain (%)	14 (100)	21 (95.5)	0.42
MMSE (/30)	25.6 ± 3.6	24.4 ± 2.4	0.32
Orientation (/10)	8.5 ± 1.6	7.8 ± 1.6	0.24
Attention (/5)	4.0 ± 1.1	3.8 ± 1.2	0.65
Memory (/6)	4.4 ± 1.4	4.0 ± 1.0	0.37
Language (/8)	7.8 ± 0.4	7.8 ± 0.4	0.82
Visualspatial (/1)	0.9 ± 0.3	1.0 ± 0.0	0.34
MoCA (/30)	18.7 ± 3.9	18.5 ± 2.8	0.86
Visualspatial and executive (/5)	3.6 ± 1.2	3.5 ± 0.9	0.86
Naming (/3)	2.7 ± 0.5	2.5 ± 0.7	0.38
Attention (/6)	5.0 ± 0.8	5.2 ± 1.0	0.58
Language (/3)	1.1 ± 0.5	1.5 ± 0.7	0.10
Abstraction (/2)	1.0 ± 0.8	1.2 ± 0.7	0.40
Delayed recall (/5)	0.3 ± 0.6	0.05 ± 0.2	0.18
Orientation (/6)	4.6 ± 1.6	4.0 ± 1.4	0.28
FAB (/18)	12.1 ± 3.0	12.4 ± 2.5	0.76
Similarities (/3)	1.8 ± 0.6	1.8 ± 0.7	0.91
Lexical fluency (/3)	2.3 ± 0.5	2.4 ± 0.7	0.47
Motor series (/3)	2.1 ± 0.8	1.5 ± 0.9	0.051
Conflicting instructions (/3)	1.4 ± 1.4	2.3 ± 1.1	0.07
Go/No-Go (/3)	1.5 ± 1.1	1.4 ± 1.0	0.76
Prehension behavior (/3)	3.0 ± 0.0	3.0 ± 0.0	1.00

MCI, mild cognitive impairment; AD, Alzheimer’s disease; aMCI, na-MCI, non-amnestic MCI; amnestic MCI; MMSE, Mini-Mental State Examination; MoCA, Montreal Cognitive Assessment; FAB, Frontal Assessment Battery.

### 3.3 Imaging-based (N) markers on MRI and SPECT and AT(N) classification

In the MCI-AD group, 18 patients (82%) were T + (Table 3), whereas no T + patients were observed in the MCI non-AD group. Thirty-four patients were classified as (N) + based on MRI or SPECT results: 14 (100%) in the MCI non-AD group and 20 (91%) in the MCI-AD group. Sixteen patients were (N) + on both MRI and SPECT (7 MCI non-AD; 9 MCI-AD). However, no significant between-group differences were observed in the overall proportion of (N) + patients or in the proportion of patients who were (N) + on both MRI and SPECT (*p* = 0.25 and *p* = 0.59, respectively).

**Table 3:** AT(N) classification of mild cognitive impairment unlikely due to Alzheimer’s disease and mild cognitive impairment due to Alzheimer’s disease.

Group (*n*)	MCI non-AD (14)	MCI-AD (22)	*p*-value
A + (%)	0 (0)	22 (100)	
T + (%)	0 (0)	18 (81.8)	
(N)+ / (N) − (%)	14 / 0 (100 / 0)	20 / 2 (90.9 / 9.1)	0.25
MRI and SPECT(N)+ / (N) − (%)	7 / 7 (50 / 50)	9 / 13 (40.9 / 59.1)	0.59
MRI(N)+ / (N) − (%)	11 / 3 (78.6 / 21.4)	8 / 14 (36.4 / 63.6)	0.013*
MTA score	2.0 ± 0.7	1.7 ± 0.5	0.13
% MTL-GM	41.3 ± 26.4	38.0 ± 25.2	0.72
% MTL ratio	7.9 ± 5.6	7.3 ± 5.4	0.75
SPECT(N)+ / (N) − (%)	12 / 2 (85.7 / 14.3)	20 / 2 (90.9 / 9.1)	0.18
Total number of ROIs with CBF reduction	2.1 ± 1.3	2.6 ± 1.7	0.31
Right parietal lobe (SD)	0.2 ± 1.2	0.9 ± 1.7	0.13
Left parietal lobe (SD)	0.3 ± 1.1	1.2 ± 2.4	0.15
Right posterior cingulate gyrus (SD)	2.5 ± 2.1	2.6 ± 1.7	0.94
Left posterior cingulate gyrus (SD)	2.7 ± 2.3	2.9 ± 1.4	0.84
Right precuneus (SD)	0.5 ± 1.0	1.0 ± 1.7	0.29
Left precuneus (SD)	0.5 ± 0.9	0.8 ± 1.3	0.42
A − T − (N) − (%)	0 (0)	0 (0)	
A − T + (N) − (%)	0 (0)	0 (0)	
A − T − (N) + (%)	14 (100)	0 (0)	
A − T + (N) + (%)	0 (0)	0 (0)	
A + T − (N) − (%)	0 (0)	0 (0)	
A + T + (N) − (%)	0 (0)	2 (9.1)	
A + T − (N) + (%)	0 (0)	4 (18.2)	
A + T + (N) + (%)	0 (0)	16 (72.7)	

MCI, mild cognitive impairment; AD, Alzheimer’s disease; MRI, magnetic resonance imaging; SPECT, single-photon emission computed tomography; MTA, medial temporal lobe atrophy; MTL, medial temporal lobe; GM, gray matter; ROI, region of interest; CBF, cerebral blood flow; SD, standard deviation. *indicates statistical significance at *p* < 0.05.

Regarding MRI findings alone, there were no significant differences between the MCI non-AD and MCI-AD groups in mean visual MTA score (*p* = 0.13), %MTL-GM (*p* = 0.72), or %MTL ratio (*p* = 0.75). According to neurodegeneration assessment using the visual MTA scale, 19 patients were regarded as MRI (N) + (11 MCI non-AD; 8 MCI-AD). The visual MTA scale showed that the proportion of MRI (N) + patients was significantly higher in the MCI non-AD group than in the MCI-AD group (*p* = 0.013).

There were no significant differences in the proportion of SPECT (N) + patients between the MCI non-AD and MCI-AD groups (*p* = 0.18) (Table 3). Furthermore, there was no significant difference in the total number of ROIs with CBF reduction (*p* = 0.31). Comparison of the degree of deviation from the normal database (expressed in SD) revealed no significant differences in regional CBF in the right and left parietal lobes (*p* = 0.13, *p* = 0.15), posterior cingulate gyrus (*p* = 0.29, *p* = 0.42), or precuneus (*p* = 0.94, *p* = 0.83).

The final AT(N) classifications in the MCI non-AD group were A − T − (N) + in all 14 patients, whereas in the MCI-AD group, 4 patients were A + T − (N)+, 2 were A + T + (N)−, and 16 were A + T + (N)+. Notably, two patients classified as (N) − were included in the MCI-AD group (Table 3).

## 4 Discussion

Among the 36 MCI patients who may be eligible for lecanemab, 61% were diagnosed with MCI-AD based on CSF biomarker testing. There was no significant difference in the overall proportion of patients classified as (N) + between the MCI non-AD and MCI-AD groups. However, when (N) + status was determined using the MRI-based MTA scale alone, the proportion of MRI (N) + patients was significantly higher in the MCI non-AD group. These findings suggest that it is challenging to identify MCI patients with AD pathology solely on the basis of conventional imaging information prior to biomarker testing.

MTA observed on structural MRI has long been recognized as an imaging biomarker for AD. A systematic review of imaging studies in AD dementia reported that brain MRI can distinguish pathologically confirmed AD from non-AD dementias with a sensitivity of 91% and a specificity of 89% (9). Furthermore, visual assessment of MTA achieves a sensitivity of 74% and a specificity of 88% (11), whereas hippocampal volumetry yields a sensitivity of 82% and a specificity of 87% in distinguishing AD dementia from healthy controls (10). Several studies have suggested that structural MRI helps identify patients with MCI who are at high risk of progression to dementia (27, 28). The predictive accuracy of visually assessed MTA appears to be independent of age, sex, educational level, MMSE score, CDR sum of boxes score, and apolipoprotein E (APOE) ε4 status (29). Additionally, volumetric measures of hippocampal atrophy have been associated with progression to dementia (10). Thus, MTA has been considered to have high sensitivity and specificity for distinguishing AD from non-AD, even at the MCI stage. However, our findings indicate that its utility may be limited in MCI patients who are potentially eligible for lecanemab. In the present study, among MCI patients who may be eligible for lecanemab, the visual MTA scale indicated MRI (N) + in 19 patients. Of these, 8 (42%) were diagnosed with AD, whereas the remaining 11 were MCI non-AD. Conversely, 14 of 17 patients (82%) with MRI (N) − had positive amyloid pathology.

Several factors may explain the reduced ability of the MTA scale to differentiate between AD and non-AD pathology in individuals with MCI who may be eligible for lecanemab. First, individuals with MCI-AD often exhibit cognitive decline before MTA becomes evident (5). Therefore, even when neuropsychological test scores are similar between groups, MCI-AD patients may still have relatively preserved medial temporal lobe volume on MRI. Moreover, MCI-AD patients who are potentially eligible for lecanemab may present to medical attention at an earlier disease stage. Second, neurodegeneration in older adults may be associated with other pathologies, such as age-related tauopathy, transactive response DNA-binding protein 43 (TDP-43) pathology, hippocampal sclerosis, and atherosclerosis (30). These conditions can also present with MTA similar to that observed in AD; however, cognitive function tends to be relatively preserved compared with AD. Third, patients with an A + biomarker profile have positive amyloid pathology; however, neurodegeneration and cognitive deficits may be attributable not only to AD but also to other comorbid pathologies. In AD, Aβ is thought to induce neuronal damage via p-tau. In individuals with an A + T − (N) + profile, both AD and non-AD pathological changes may contribute to neurodegeneration and cognitive impairment (5).

No significant differences were observed between the MCI non-AD and MCI-AD groups in the proportion of SPECT (N) + cases, the degree of CBF reduction within ROIs (parietal lobe, posterior cingulate gyrus, or precuneus), or the total number of regions with CBF reduction. A meta-analysis of 20 studies evaluating CBF changes in AD dementia using SPECT reported that regional hypoperfusion in the temporal, parietal, and limbic lobes is associated with cognitive decline (12). However, in a multicenter prospective cohort study investigating the utility of ^123^I-IMP SPECT in MCI, automated ROI analysis of the parietal lobes and posterior cingulate cortex predicted progression to AD dementia within 3 years with a relatively low accuracy of 58% (sensitivity, 81%; specificity, 37%) (31). Previous studies have demonstrated that SPECT is useful for distinguishing AD dementia from healthy controls. At the MCI stage, however, its accuracy for differentiating AD from non-AD is modest, particularly with respect to specificity, indicating a high likelihood of false positives among MCI non-AD patients. In our study, 90.9% of patients with MCI-AD were SPECT (N)+, while 85.7% of those with MCI non-AD were also SPECT (N)+. These findings indicate that distinguishing AD from non-AD in MCI patients who may be eligible for lecanemab using SPECT alone is also challenging. Moreover, two patients who were (N) − on both MRI and SPECT nevertheless exhibited positive AD pathology, underscoring the need for caution: negative imaging-based (N) markers do not necessarily exclude underlying AD pathology in lecanemab-eligible MCI patients.

In this study, we selected CSF biomarkers rather than amyloid PET to assess AD pathology. CSF biomarkers quantify the concentrations of specific proteins and reflect the dynamic processes of protein production (expression or release from neurons or other brain cells) and clearance (degradation or removal) at a given time (5). In contrast, amyloid PET imaging reflects the magnitude of neuropathological burden that has accumulated over time (32). Consequently, low CSF Aβ42 levels are best interpreted as an indicator of a pathological state associated with amyloid plaque formation, rather than as a direct measure of plaque burden, which is better captured by amyloid PET. CSF biomarkers are therefore well suited for detecting amyloid pathology at the MCI stage, which represents an early phase of AD (33). Measurement of the Aβ42/Aβ40 ratio provides greater accuracy in detecting AD pathology than CSF Aβ42 levels alone (34). In a study using Fujirebio’s Lumipulse® system, the Aβ1-42/Aβ1-40 ratio, with a cutoff of 0.067, was evaluated in patients with MCI due to AD, patients with mild AD dementia, and controls. Concordance between the Aβ1-42/Aβ1-40 ratio and amyloid PET was assessed, demonstrating a sensitivity of 81.9%, specificity of 81.9%, and overall accuracy of 81.9%, highlighting the diagnostic reliability of this ratio as a biomarker for amyloid pathology (35). Nevertheless, compared with amyloid PET, CSF biomarkers have several disadvantages. Although CSF testing is less costly than amyloid PET, it requires specialized facilities and expertise in lumbar puncture and sample processing. Lumbar puncture is invasive and may cause discomfort and complications such as back pain or headache, which can discourage patients from undergoing the procedure. Furthermore, CSF biomarkers are susceptible to variability introduced by pre-analytical factors, including sample handling, storage conditions, and differences between assay platforms, which may reduce reproducibility across laboratories. Accordingly, lumbar puncture is not routinely performed in all individuals with MCI. It is generally reserved for patients whose clinical features suggest AD, and is performed after careful explanation of potential risks and adverse effects.

This study has several limitations. First, the small sample size (36 patients) may limit the generalizability of the findings and reduce statistical power, particularly for subgroup analyses distinguishing MCI-AD from MCI non-AD. Second, we included only MCI patients who may be eligible for lecanemab treatment. Patients with cerebral microbleeds, cortical superficial siderosis, or large cerebral hemorrhages were excluded. Thus, our findings are not generalizable to the broader MCI population, nor to the differentiation of AD and non-AD etiologies in unselected MCI cohorts. Third, CSF biomarker testing requires an invasive lumbar puncture, which may deter patient participation. Some participants underwent amyloid PET rather than CSF testing for biomarker confirmation, and some patients with MCI who may have been eligible for lecanemab could not be enrolled. Fourth, we used SPECT rather than FDG-PET to assess neurodegeneration. Although SPECT is widely used in clinical practice in Japan, it is not recognized as an official (N) marker under NIA–AA guidelines, which may affect the reliability and validity of our neurodegeneration assessments. Finally, we did not include APOE genotyping in the prediction of AD pathology. APOE genotype provides crucial information that can support the diagnosis of AD. AD is a multifactorial disease with a substantial genetic component, and APOE is the strongest common genetic risk factor (36). The ε4 allele confers a markedly increased risk of developing AD and of progression from MCI to AD dementia (37). The risk of AD is approximately 3.2-fold higher in ε4 heterozygotes and 11.6-fold higher in ε4 homozygotes than in individuals with an ε3/ε3 genotype (38). These findings suggest that incorporating genetic information alongside imaging-based (N) markers could improve the accuracy of predicting AD pathology in MCI patients who may be eligible for lecanemab.

## 5 Conclusion

**Figure 2:**
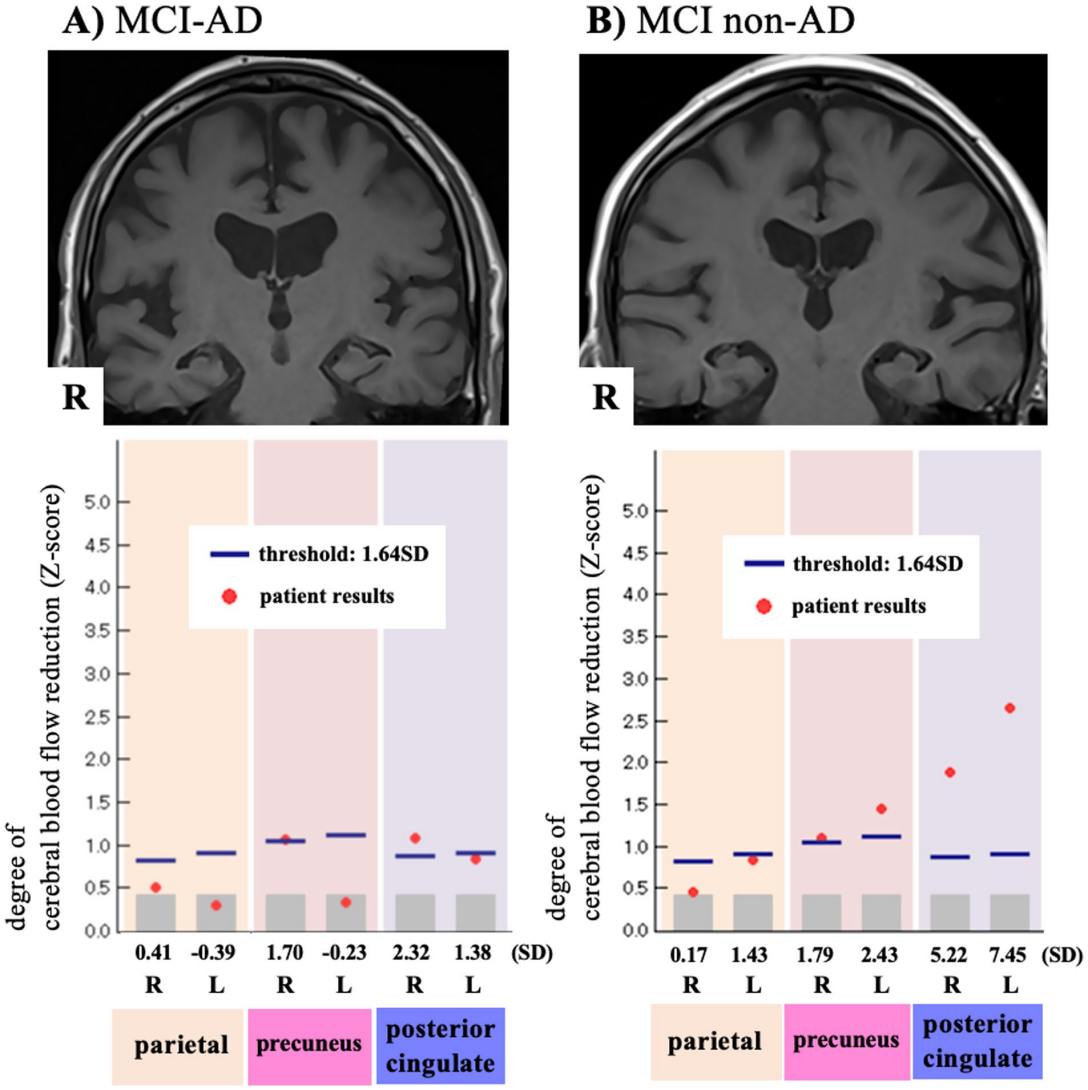
Example of medial temporal lobe atrophy and cerebral blood flow reduction in mild cognitive impairment with and without Alzheimer’s disease. **(A)** 79-year-old woman with MCI-AD [A + T + (N)+] and **(B)** 77-year-old man with MCI non-AD [A-T-(N)+]. Both have medial temporal lobe atrophy (top row) and cerebral blood flow reduction (bottom row) in the precuneus and posterior cingulate gyrus, which making it difficult to distinguish AD from non-AD using the imaging-based (N) marker alone. MCI, mild cognitive impairment; AD, Alzheimer’s disease; SD, standard deviation.

Our findings highlight that relying on imaging-based (N) markers when deciding whether to perform CSF biomarker testing in MCI patients who may be eligible for lecanemab can reduce diagnostic efficiency for AD and may prevent the identification of patients who could benefit from anti-Aβ therapy. Conversely, even when MRI or SPECT findings suggest AD pathology, overinterpretation should be avoided until biomarker testing results are available (Figure 2). The development and insurance coverage of non-invasive blood-based biomarkers may enable direct confirmation of AD pathology, eliminating the need for preliminary screening using imaging-based (N) markers. A recent validation study demonstrated that clinical signs such as the head-turning sign exhibit high specificity and positive predictive value for amyloid and tau PET positivity and are significantly correlated with established AD-related blood biomarkers (39). These findings suggest that widely recognized, simple behavioral signs may facilitate early screening. When screening is required prior to CSF biomarker testing, a comprehensive assessment incorporating clinical signs, rather than reliance solely on imaging, is warranted.

## Data Availability

The raw data supporting the conclusions of this article will be made available by the authors, without undue reservation.
